# Transcellular Targeting of Fiber- and Hexon-Modified Adenovirus Vectors across the Brain Microvascular Endothelial Cells *In Vitro*

**DOI:** 10.1371/journal.pone.0045977

**Published:** 2012-09-27

**Authors:** Johanna P. Laakkonen, Tatjana Engler, Ignacio A. Romero, Babette Weksler, Pierre-Olivier Couraud, Florian Kreppel, Stefan Kochanek

**Affiliations:** 1 Department of Gene Therapy, University of Ulm, Ulm, Germany; 2 Inserm, U1016, Cochin Institute, Paris, France; 3 CNRS, UMR8104, Paris, France; 4 University Paris Descartes, Paris, France; French National Centre for Scientific Research, France

## Abstract

In central nervous system (CNS)-directed gene therapy, efficient targeting of brain parenchyma through the vascular route is prevented by the endothelium and the epithelium of the blood-brain and the blood-cerebrospinal fluid barriers, respectively. In this study, we evaluated the feasibility of the combined genetic and chemical adenovirus capsid modification technology to enable transcellular delivery of targeted adenovirus (Ad) vectors across the blood-brain barrier (BBB) *in vitro* models. As a proof-of-principle ligand, maleimide-activated full-length human transferrin (hTf) was covalently attached to cysteine-modified Ad serotype 5 vectors either to its fiber or hexon protein. In transcytosis experiments, hTf-coupled vectors were shown to be redirected across the BBB models, the transcytosis activity of the vectors being dependent on the location of the capsid modification and the *in vitro* model used. The transduction efficiency of hTf-targeted vectors decreased significantly in confluent, polarized cells, indicating that the intracellular route of the vectors differed between unpolarized and polarized cells. After transcellular delivery the majority of the hTf-modified vectors remained intact and partly capable of gene transfer. Altogether, our results demonstrate that i) covalent attachment of a ligand to Ad capsid can mediate transcellular targeting across the cerebral endothelium *in vitro*, ii) the attachment site of the ligand influences its transcytosis efficiency and iii) combined genetic/chemical modification of Ad vector can be used as a versatile platform for the development of Ad vectors for transcellular targeting.

## Introduction

The endothelial cells of the blood-brain barrier (BBB), lining the inner surface of the brain capillary endothelium, form the major barrier to the passage of macromolecules, circulating cells and pharmaceutical drugs from blood to brain parenchyma. To develop treatments for central nervous system (CNS) disorders such as Multiple Sclerosis, Alzheimer’s or Parkinson’s disease, numerous studies have attempted to enhance drug or vector delivery across the BBB by targeting receptor molecules residing on the luminal side of the brain microvascular endothelial cells known to be involved in transcytosis [1], [2]. Previously, various *in vitro* BBB models have been used as valuable tools to estimate the potency of the drugs or vectors to cross the brain endothelium [3]. *In vivo*, receptor-mediated transcytosis across the BBB has been suggested with molecules such as iron-transferrin, melanotransferrin, insulin, TNF-alpha and leptin [1], [2], [4] and absorptive or fluid-phase transcytosis with molecules such as albumin, immunoglobulin G, wheat germ agglutinin and avidin [5]. The transcellular delivery and internalization of vectors based on viruses, antibodies, protein carriers, liposomes, or nanoparticles has been shown to be dependent on various parameters such as their solubility, size, charge and receptor-ligand interactions [2], [6].

Human adenovirus serotype 5 vectors (Ad5) are among the most efficient gene transfer vectors available for CNS-directed gene therapy into non-dividing cells [7]–[9]. In brain tissue, long-term Ad-mediated gene expression [10] and transcriptional targeting by tissue specific promoters [11], [12] have previously been demonstrated after intracranial injection. Besides inflammatory responses upon vector administration into the brain [11], [13], [14], small injection volumes in stereotactic delivery as well as poor spreading of the Ad vectors in brain parenchyma have limited the development of gene therapy strategies for neurological disorders. While many studies have shown successful transduction of peripheral endothelial cells and unpolarized cerebral endothelial cells by tropism-modified Ad vectors [15], [16], Ad-mediated gene delivery in or across confluent, polarized cerebral endothelial cells has received less attention. Recently, for the first time, tropism-modified Ad vectors were shown to be targeted across bovine brain microvascular endothelium by LRP-receptor mediated transcytosis [17], showing that the size of Ad vectors did not inhibit the transcellular delivery *in vitro*. To date, no *in vitro* studies of targeted Ad delivery across human brain microvascular endothelium by transcytosis have been presented.

Previously, we demonstrated genetic introduction of cysteine residues into the Ad fiber, hexon or pIX proteins enabling covalent attachment of maleimide-activated ligands to defined sites of the Ad vector capsid [18]–[20]. In this study, we utilized the cysteine-based targeting platform of Ad vectors to analyze whether Ad5 vectors could be targeted across polarized brain microvascular endothelium *in vitro* by using a known transcytotic ligand, human transferrin. Despite of transductional targeting of the Ad vectors to non-polarized brain microcapillary endothelial cells by transferrin receptor (TfR) -targeted peptide motifs (Xia et al. 2000), no previous studies have been performed in polarized BBB cell models, which have unique receptor patterns on their apical/basolateral side, restricted paracellular passage and transcellular delivery mechanisms. In the present study, we demonstrate that i) transductional targeting of Ad vectors in the cerebral endothelial cells is dependent on the cellular integrity of the polarized brain microvascular endothelium, ii) Ad vectors do not disrupt the brain microvascular endothelium integrity, iii) transferrin-receptor targeted Ad vectors can be delivered across the endothelial cell barrier *in vitro*, and that iv) majority of the targeted vectors remain intact after their transcellular delivery. In summary, we demonstrate that combined genetic/chemical modification of Ad vectors may be used as a platform for development of Ad vectors with improved transcytosis activity.

## Results

### Unpolarized Human Brain Microvascular Endothelial Cells are Highly Susceptible to Transferrin-Coupled Ad Vectors

**Figure 1:**
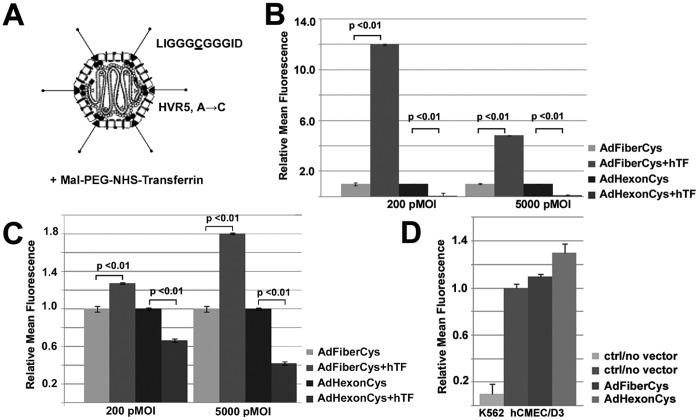
Covalent attachment of maleimide-activated human transferrin to cysteine-modified Ad vectors and their transduction efficiency in hTfR-positive human brain microvascular endothelial cells. A) Schematic illustration of Ad vector particles containing a solvent-exposed cysteine either on fiber (LIGGGCGGGID) or hexon (HRV5, alanine to cysteine substitution), to which maleimide-activated transferrin is covalently attached (for details see materials and methods). B–C) Relative transduction efficiency of fiber (AdFiberCys) and hexon-modified (AdHexonCys) vectors with or without covalently attached transferrin in K562 (B) and hCMEC/D3 (C) cells at 24 hrs p.t. by flow cytometry (multiplicities of infection based on particles (pMOIs) 200 and 5000). Relative mean fluorescence and standard deviations are shown (n = 3, 10.000 cells/sample). D) Cellular uptake of the fluid-phase endocytosis marker 70 kDa FITC-Dextran in untransduced K562 and hCMEC/D3 and transduced hCMEC/D3 cells (pMOI 5000) after 1 hr uptake at 37°C determined by flow cytometry. Relative mean fluorescence normalized to untransduced hCMEC/D3 cells, as well as standard deviations are shown (n = 3, 10.000 cells/sample).

Previously, we demonstrated the successful production and transductional targeting of cysteine modified Ad vectors [18]–[20]. In this study, transferrin coupled cysteine-bearing Ad vectors were utilized for transcellular targeting across the brain microvascular endothelium *in vitro*. To chemically couple human transferrin (hTf) to cysteine-modified Ad vectors, maleimide activation of hTf was performed with a heterobifunctional crosslinker, followed by coupling of activated hTf to thiol groups of the fiber HI loop (AdFiberCys) or the hypervariable region 5 of hexon (AdHexonCys, Fig. 1A). Successful coupling was confirmed by western blot analysis and chemiluminescence detection by Ad fiber or hexon antibodies (data not shown) as previously presented [19], [20].

To detect the transductional targeting of hTf-coupled Ad vectors, vector-mediated EGFP expression was analyzed by flow cytometry in the immortalized human brain microvascular endothelial cells (hCMEC/D3) and in the non-endothelial human hematopoietic (K562) cell line, known to express the human transferrin receptor (hTfR) on their surface [21], [22]. Flow cytometry experiments at 24 hrs post transduction (p.t.) in semiconfluent, unpolarized hCMEC/D3 cells showed that hTfR-targeted fiber-modified vectors had a 1.3 and 1.8-fold higher transduction efficiency than the untargeted control vectors at pMOIs of 200 and 5000 (expression percentages 33.8% and 75.4%, Fig. 1C, *P*<0.01), resulting in increased transgene expression in 62.9%±0.92 and 94.3%±0.31 of cells, respectively (Fig. 1C, *P*<0.01). In contrast, hTf-coupled hexon-modified vectors had a 1.5-fold and 2.4-fold lower transduction efficiency (19.5% and 80.9%) than corresponding untargeted control vectors (48.6% and 94.3%, Figs. 1C, *P*<0.01). In line with our previous studies (Kreppel et al. 2005), EGFP transgene expression of hTfR-targeted fiber-modified vectors increased up to 12-fold in K562 cells (41.3% and 94.9%, Fig. 1B) in comparison to AdFiberCys transduced cells (9.58% and 55.8%). The transduction efficiency of hTfR-targeted hexon-modified vectors instead decreased by 12 to 20-fold (1.2% and 19.0%, Fig. 1B) compared with their corresponding untargeted control vectors (17.9% and 68.0%).

Since the transduction efficiency of unpolarized hCMEC/D3 cells with hTfR-targeted hexon-modified Ad vectors was significantly higher than in K562 cells (pMOIs 1000–5000), we hypothesized that unspecific fluid-phase endocytosis could be involved in the uptake of the vectors in hCMEC/D3 cells. Therefore, the activity of unspecific fluid-phase endocytosis in the absence or presence of Ad vectors was determined by flow cytometry using 70 kDa FITC-dextran. After 1 hr uptake, untransduced hCMEC/D3 cells were detected to have 10-fold more active internalization of dextran than the corresponding untransduced K562 cells (Fig. 1D). However, no significant change of dextran uptake was detected in the presence of untargeted Ad vectors at pMOIs of 200 and 5000, respectively (*P*>0.1, Fig. 1D, pMOI 5000; pMOI 200, data not shown). In addition to dextran uptake, expression of hCAR, the primary receptor of Ad5, was determined in unpolarized hCMEC/D3 cells by immunolabeling experiments. By flow cytometry, hCAR expression was detected in 34.4%±2.34% of hCMEC/D3 cells (data not shown), whereas expression has been shown to be nearly non-existent in K562 cells [23]. Altogether, these data suggest that in unpolarized hCMEC/D3 cells the uptake of fiber-modified Ad vector is in part mediated by the transferrin receptor. However, regardless of the TfR-targeting, fluid-phase endocytosis may also contribute to the uptake of all the vectors in hCMEC/D3 cells but is not further enhanced by high vector doses. Additionally, hCAR-mediated entry of hexon-modified vectors may increase their uptake into hCMEC/D3 cells. Since both vector types expressed their transgenes efficiently in hCMEC/D3 at high pMOIs, the decreased transduction efficiencies of hexon-modified vectors in K562 cells (pMOIs 200–5000) is likely due to aberrant intracellular trafficking of the vectors [24], [25].

### Transduction Efficiency of Brain Microvascular Endothelial Cells is Dependent on the Cellular Integrity of the Polarized Endothelium

**Figure 2:**
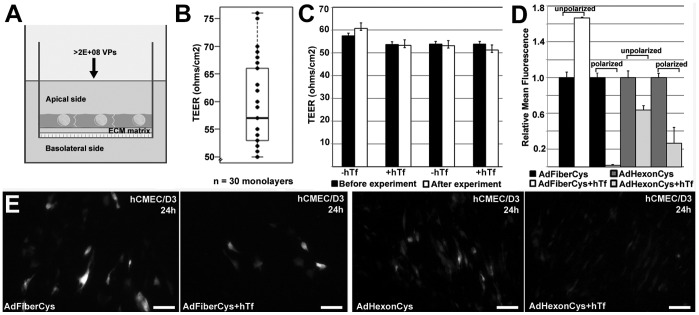
Integrity of hcmec/d3 endothelium in the presence of Ad vectors and their transduction efficiencies in polarized cells. A) Schematic illustration of the *in vitro* hCMEC/D3 endothelium model on collagen-coated 0.4 µm-Transwell filters. Cells are grown to confluency for 6–7 days in EBM-2 media, after which the barrier properties of the endothelium are measured by voltohmeter and permeability assays with fluorescent markers. Extracellular matrix (ECM), filter, and apical and basolateral sides of the Transwell chamber are shown. B) Transendothelial electrical resistance (TEER) values of hCMEC/D3 monolayers after 6 days in culture (59.4±1.5 Ω/cm^2^; mean±S.E., n = 30 monolayers). Boxplot data is shown, containing median (bar), quartile range (box) and minimum and maximum values (whiskers). C) Representative TEER values of hCMEC/D3 monolayers before and after 4 hrs incubation with hexon (AdHexonCys, 5×10^9^ VPs/monolayer) or fiber-modified (AdFiberCys, 1×10^9^ VPs/monolayer) vectors with or without human transferrin (n = 3–4 monolayers/vector). In all experiments, TEER measurements were performed as triplicates with Milli-Cell ERS equipment (mean Ω/cm^2^±SD, *P*>0.1). D–E) Transduction efficiencies of fiber or hexon-modified vectors with or without transferrin in unpolarized or transwell-cultured, polarized hCMEC/D3 cells at 24 hrs p.t. detected by fluorimetry (D; n = 3 monolayers, TEER >60 Ω/cm^2^) or fluorescence microscopy (E; polarized cells). Relative mean fluorescence and standard deviation is calculated from the obtained mean fluorescence values. Scale bars in the images, 50 µm.

To study the transductional and transcellular targeting into/across the endothelium barrier *in vitro*, two-compartment endothelial cellular models are required. Here, hCMEC/D3 cells cultured on collagen-coated Transwell-filters were used to determine the targeting ability of hTf-coupled Ad vectors. Previously, hCMEC/D3 cells have shown to retain many characteristics of primary brain capillary endothelial cells and to form a highly restrictive endothelium barrier [26]. In this study, for transcytosis and transduction experiments in polarized cells, hCMEC/D3 cells were grown for 6–7 days on a Transwell system with an apical chamber containing a collagen-coated insert (pore size 0.4 µm, apical side) and a bottom, basolateral chamber (Fig. 2A). Prior to experiments with virus vectors, the cell monolayer integrity was analyzed by measuring the passage of small ions i.e. transendothelial electrical resistance (TEER) or diffusion of fluorescent markers across the endothelium. TEER was measured from each well as a triplicate with Endohm Millicell device. In hCMEC/D3 endothelial monolayers, all transcytosis experiments were performed at TEER values 50–75 Ω/cm^2^ (Fig. 2B). In addition, permeability coefficient for lucifer yellow (LY) and 70 kDa FITC-Dextran was determined. All obtained permeability values were shown to be in the range of published permeability index values (Poller et al. 2008) i.e. 0.81×10^−3^ cm/min for LY and 0.05×10^−3 ^cm/min for FITC-Dextran (n = 3 monolayers), resembling the tight characteristics of the monolayers. To detect the impact of Ad vectors on the hCMEC/D3 barrier integrity and functionality, TEER was measured before and after transcytosis experiments. The resistances of the monolayers were unchanged during the 4 hr-experiments with all vector types and particle amounts (Fig. 2C, *P*-values >0.1).

To assess the vector transduction efficiency in polarized brain endothelium, known to express TfR only on their apical side, hCMEC/D3 cells were cultured on Transwell filters to confluency for 6–7 days (TEER >50 Ω/cm^2^) and transduced for 24 hrs with Ad vectors. By fluorescence microscopy, the percentage of cells expressing EGFP was visibly lower with all the vectors than in unpolarized hCMEC/D3 cells without the barrier properties (data not shown). By further analysis with fluorimetry, uncoupled fiber-modified Ad vectors were shown to transduce polarized endothelium more efficiently than the corresponding hTf-coupled vectors (Fig. 2D, *P*<0.01). This suggests that in polarized cells hTf-coupled Ad vectors had a limited accessibility to TfRs or that the vectors were redirected to a different cellular route than in non-polarized endothelial cells.

### Transferrin-Receptor Targeted Ad Vectors are Delivered Across the Brain Endothelial Barrier

To determine the ability of hTf-targeted Ad vectors to be transported across the endothelium barrier, the experiments were performed in the hCMEC/D3 Transwell cell model. Unmodified or modified Ad vectors (2×10^8^, 1×10^9^ or 5×10^9^ VPs) were added to Transwell plates for 4 hrs and the medium from the basolateral side was collected. To validate the copy numbers of transcytosed viral particles, real-time quantitative PCR (qPCR) for the Ad fiber or E4 gene was performed. The cellular barrier integrity was monitored by measuring TEER before and after incubation with Ad vectors to confirm that vector delivery to the basolateral side was not due to leakage of the endothelial barrier.

**Figure 3:**
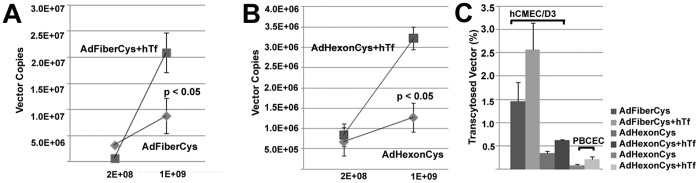
Delivery of transferrin-coupled fiber-modified Ad vectors across the endothelium barrier. A, B) After transcytosis experiments in Transwell plates, qPCR was performed from the basolateral media using Ad fiber and E4 primers. The corresponding Ad copy number was determined by the standard curve of linearized pGS66 plasmid. The detected vector copy numbers of fiber- (A) and hexon-modified vectors (B) are shown (2×10^8^, n = 2–3 monolayers; and 1×10^9^ VPs, *P*<0.05, n = 6 monolayers; mean ± S.E.). C) Transcytosis percentages of the vectors after transcellular delivery detected by qPCR across hCMEC/D3 and PBCEC cells (1×10^9^ and 5×10^9^ VPs). The percentages were calculated by comparing the detected copy numbers of the input vector to the copy numbers on the basolateral side.

With hTf-coupled fiber-modified vectors, 2.4-fold increase in transport across the polarized hCMEC/D3 cells was observed in comparison to untargeted control vectors (Fig. 3A, 3C, p<0.05, 1×10^9^ VPs, 2.57%; n = 6 monolayers). The copy numbers of vectors detected on the basolateral side increased with higher vector dose. Interestingly, with a lower vector dose (2×10^8^ VPs), more untargeted vector (AdFiberCys) was detected on the basolateral side than with TfR-targeted vectors (*P*<0.01, n = 3 monolayers), implying that the vector uptake, recycling to apical side or delivery mechanisms across the cell monolayer differed between the targeted and untargeted vectors. Notably, no significant changes in TEER values were detected between 0 to 4 hrs after adding the Ad vectors, indicating that the hCMEC/D3 cells maintained a functional barrier (data not shown).

With hexon-modified vectors, re-targeting of hTf-coupled vectors was also detected at higher vector dose (Fig. 3B, *P*<0.05, 1×10^9^ VPs, n = 6 monolayers). However, overall percentage of the targeted transcytosed vectors remained low (0.63%, Fig. 3C). No targeting with lower vector dose was detected (2×10^8^ VPs, n = 3 monolayers). To analyze the transcytosis capability of hTf-modified Ad vectors further and determine their ability to be used in CNS-directed targeting in general, another BBB cell model based on primary porcine brain capillary endothelial cells (PBCEC) was used. The PBCEC model is known to have one of the highest TEER values *in vitro* and a very low cell monolayer permeability [27]–[29]. Here, all experiments in PBCEC cells were performed as TEER reached >600 ohms/cm^2^. With hTf-targeted fiber-modified vectors, no reliable vector copy amounts were detected by qPCR either with E4 or fiber primers, due to the extremely low vector presence in the basolateral media (data not shown, n = 3 monolayers). However, a 3.7-fold increase of hTf-targeted AdHexonCys delivery across the cell monolayer was detected (Fig. 3C, 0.21%, 5×10^9^ VPs, n = 2–3 monolayers).

### Part of the Re-targeted Ad Vectors Remains Capable of Gene Transfer after Transcellular Targeting

**Figure 4:**
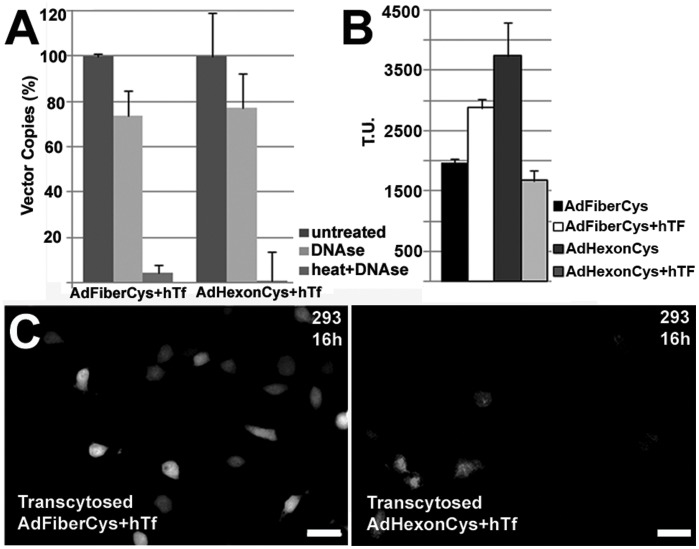
Delivery of transferrin-coupled hexon-modified Ad vectors across the endothelium barrier. A) qPCR detection of the transcytosed hTf-coupled vectors after DNAse treatment and viral DNA isolation. Heat and DNAse treated vectors, as well as untreated vectors were used as controls. The percentages were calculated by comparing the detected copy numbers of the untreated vector to the copy numbers of the transcytosed vector. B–C) Gene transfer efficiency of transcytosed hTf-coupled vectors in 293 cells at 16 hrs p.t. as determined by fluorescence microscopy. For quantification, six to eight random areas were imaged and the cells were counted with the help of ImageJ Cell Counter. Transduction units (T.U.) presented were counted from the total sample volume obtained on the basolateral side of the hCMEC/D3 cells (n = 500 cells, mean ± S.E.). Scale bars in all images, 20 µm.

To test whether transferrin-modified vectors remained intact and DNAse resistant after delivery, transcytosed vectors were treated with heat and/or DNAse prior to virus DNA extraction and analysis by qPCR. In comparison to non-treated transcytosed vectors, the majority of heat and DNAse treated transcytosed vectors were degraded (Fig. 4A), whereas DNAse treatment alone decreased the transcytosed viral DNA detection by only 1.3 to 1.5-fold. The data therefore indicated that the majority of the vector DNA remained encapsulated and DNAse resistant after the transcellular delivery.

Previously, cellular uptake of hTf across the BBB has been shown to occur by receptor-mediated endocytosis, followed by the release of iron-transferrin to brain parenchyma [30]. To determine whether transcytosis and endosomal delivery also affected the gene transfer capability of transcytosed Ad vectors, transduction studies in hTfR positive 293 cells were performed using the basolateral media collected from the Transwell chambers after transcytosis. By fluorescence microscopy vector-mediated EGFP expression was detected at 16 hrs p.t., showing that some of the transcytosed Ad vectors remained functional and the cellular processes did not harm the integrity of the vectors (Fig. 4B–C). hTf-coupled fiber-modified vectors were shown to have approximately an 1.5-fold increase in their transgene expression compared to unmodified control vectors after their transcellular delivery (Fig. 4B, n = 500 cells). No transgene expression was detected in the corresponding experiments with the PBCEC cells (data not shown). With the hexon-modified vectors hTf-coupling led to a 2.3-fold decrease in EGFP transgene expression after their transcellular delivery across hCMEC/D3 (Figs. 4B–C, n = 400–500 cells) and 4.1-fold decrease in PBCEC cells, respectively (data not shown).

## Discussion

In brain disorders, inefficient cellular targeting and distribution of therapeutic macromolecules have been restricting the development of brain-related gene therapy strategies. Since only <2% of small drug molecules are able to cross the BBB after systemic administration and large molecules are believed not to pass the BBB at all [31], CNS-directed gene therapy has mainly been based on intracranial injections [9]. Besides the invasive surgical procedures, numerous localized injections are typically needed for sufficient gene delivery. Since most of the CNS diseases are currently poorly or not treatable with small molecule therapies [32], development of gene therapy vectors suitable for overcoming the BBB non-invasively upon systemic administration has been suggested [17], [33]. In the present study, we evaluated the feasibility of combined genetic and chemical Ad capsid modification to target Ad vectors across the BBB *in vitro* by transcytosis. As a proof-of-principle ligand we used human transferrin, a well characterized transcytotic ligand which has been shown to bind TfR on the luminal side of the brain endothelial cells and to be delivered to brain parenchyma after receptor-mediated uptake [1], [30]. In this paper, we showed that transferrin led to enhanced transcellular delivery of fiber- and hexon-modified Ad vectors across the polarized brain endothelium *in vitro*, the transport being approximately 2 to 4-fold higher than that of untargeted control vector. Importantly, the targeted vectors stayed largely intact and partly functional after transcytosis, showing the potential of the combined genetic and chemical modification of Ad vectors in successful transcellular targeting.

*In vitro* cerebral endothelium models have been shown to be valuable tools for development of pharmacological drug delivery and gene therapy, being solely based on primary or immortalized brain microvascular endothelial cells or cocultures of astrocytes and/or pericytes. In this study, we used two *in vitro* BBB models, immortalized hCMEC/D3 and primary PBCEC cells, which have been shown to form highly restrictive endothelium barriers and to have similar permeability characteristics than primary human brain microvascular endothelial cells [21], [26]–[28], [34]. Similarly as in previous studies with the bovine brain endothelium with untargeted Ad5 [35] or melanotransferrin-targeted Ad5 vectors [17], [35], no significant changes of TEER were observed in the presence of Ad vectors with or without transferrin, thus implying that the Ad vectors did not alter the interendothelial integrity of the cerebral endothelium. Interestingly, despite of the high TfR expression on the apical side of polarized hCMEC/D3 cells [21] and transgene expression in unpolarized hCMEC/D3 cells (20–60% efficiencies, 200 pMOI), poor transduction efficiencies of iron-transferrin targeted Ad vectors were detected in polarized hCMEC/D3 cells. This strongly indicated that in polarized brain endothelium, the majority of iron-transferrin targeted Ad vectors were redirected to another cellular entry route leading either to recycling of vectors back to the apical side, lysosomal degradation or transcellular delivery of the vectors across the endothelium.

For gene therapy purposes, virus vectors need to stay intact and capable of gene transfer after their transcellular delivery. Recently, systemic administration of rAAV9 was shown to lead to transduction of astrocytes in adult mice (Foust et al. 2009). Additionally, *in vitro* transcellular delivery of HIV-1 (0.01–0.77% at 24 hrs p.t), AAV4, AAV5 or BAAV (3–15% at 3 hrs p.t) and melanotransferrin-targeted Ad5 (5% at 6 hrs p.t.) have been detected to cross the BBB *in vitro* models, simultaneously retaining their infectivity or capability for gene transfer [35], [36]. In this study, targeting of Ad5 by iron-transferrin led to enhanced transcytosis of fiber- and hexon-modified vectors. The targeting across the endothelium was shown to be dose-dependent, being characteristics of receptor-mediated uptake. With hexon-modified Ad vectors, transcytosis percentage remained modest (0.6% at 4 hrs p.t.), whereas 2.6% of fiber-modified vectors were able to cross the human *in vitro* BBB model. The experiments in primary porcine cerebral endothelium showed transport of 0.2% of the hexon-modified Ad vectors across the cells and no transcellular targeting of the fiber-modified vectors, implying that the mode and activity of transcellular delivery vary between immortalized and primary brain microvascular endothelial cells. Similar to studies with untargeted AAV viruses [35] the majority of the TfR-targeted Ad vectors were shown to remain intact after their transcellular delivery. Additionally, transcytosed vectors were able to mediate transgene expression. The delivery of the vectors across the brain endothelium in transcellular vesicles thus did not seem to impair the majority of the capsids. Notably, as in previous Ad vector studies [17] non-specific crossing of the endothelium by untargeted vectors was observed, probably due to undetected paracellular leakage. In our study, human brain microvascular endothelium seemed to be more permeable to human Ad5 vectors than primary porcine cells were, which might be due to species specific differences between the human and non-human brain endothelium or differences between immortalized and primary brain microvascular endothelial cells, such as tightness of the cell monolayer, variations in receptor expression patterns or differences in the mode and activity of transcellular delivery mechanisms. However, as Ad vectors were clearly targeted by iron-transferrin across the human brain microvascular endothelium without affecting the TEER, we can conclude that hCMEC/D3 cell model is suitable for screening ligands for Ad vector mediated transcellular targeting.

In order to circumvent the BBB and to deliver therapeutic macromolecules to brain parenchyma, multiple delivery approaches have been attempted, e.g. intrathecal injection to the cerebrospinal fluid, intracisternal administration to ventricles, intranasal delivery and intracarotid infusion of hyperosmolar solutions or vasoactive drugs. Restricted diffusion of the vector and cerebral edema due to the disrupted BBB has been the limiting factors of these approaches. Numerous factors have also been shown to affect the vectors targeted to cross the BBB by receptor-mediated transcytosis after intravenous administration, such as competitive receptor binding with endogenous ligands, irreversible antibody-antigen binding, species-specific differences between receptor expression patterns, charge of the target ligand, high expression of target receptors in the peripheral capillaries and immune reactions against the vectors [1], [6]. So far very little has been known about the transcellular targeting of large particles such as gene therapy vectors across the human microvascular brain endothelial cells. While our data demonstrate the general feasibility of Ad targeting *in vitro*, our model ligand is likely to be suboptimal for *in vivo* purposes due to competition of receptor binding by the endogenous transferrin and Ad vector targeting to liver after intravenous administration. Therefore, our Ad capsid modification technology will be used to study the transcellular targeting of novel transcytotic ligands derived from bacteria or viruses, which have been suggested to be involved in the transcellular delivery of large pathogens across the BBB *in vivo* [37]. In regard to *in vivo* transcellular targeting of Ad5 vectors, hexon based PEGylation combined with coupling of transcytotic ligands to the Ad fiber may be an option to enhance Ad5 vector targeting across anatomical barriers, as polymer coating may decrease capsid interaction with cellular or non-cellular compartments.

To conclude, our data indicate that i) virus vectors can be targeted across the brain microvascular endothelial cells *in vitro* by chemically attaching transcytotic ligands on their capsid surface, ii) full-length protein attached to the Ad vector surface is capable of mediating transcellular targeting *in vitro* and that iii) attachment site of the ligand on Ad capsid affects to the transcytosis efficiency of the vectors. This study also suggests that combined genetic/chemical modification of Ad vector with targeting ligands can be used as a versatile platform for the development of Ad vectors with improved transcytosis activity.

## Materials and Methods

### Cells and Cell Barrier Models

Human embryonic kidney cells (293, ATCC CRL-1573) and lung carcinoma epithelial cells (A549, ATCC CCL-185) were cultured in MEM, the human erythroleukemia cell line K562 (ATCC CCL-243) in RPMI-1640 and human N52.E6 cells (Schiedner et al. 2000) in α-MEM (Invitrogen, Eugene, OR). All cell lines were supplemented with 10% fetal calf serum (FCS) and 1% penicillin/streptomycin (Invitrogen) and subcultured twice a week.

The human BBB *in vitro* model based on immortalized human brain capillary endothelial cells (hCMEC/D3) has been described previously [26]. Briefly, hCMEC/D3 cell line was generated by isolating brain microvascular endothelial cells from the human brain tissue, followed by immortalization with catalytic subunit of human telomerase and SV40-T antigen. hCMEC/D3 cells used in the experiments of this report were between passages 25 to 35, and were routinely cultured on plates coated with rat-tail collagen type I (0.15 mg/ml; Cultrex, R&D Systems Inc., Minneapolis, MN) at a cell density of 27.000 cells/cm^2^. The cells were grown in the endothelial basal medium (EBM-2; Clonetics, Cambrex BioScience, Wokingham, UK) supplemented with human basic fibroblast growth factor (bFGF, 1 ng/ml), hydrocortisone (1.4 µM), ascorbic acid (5 µg/ml; Sigma-Aldrich, St. Louis, MO), penicillin-streptomycin (1%), chemically defined lipid concentrate (1%; Invitrogen) and FBS Gold (5%; PAA Laboratories GmbH, Pasching, Austria). For transwell assays, hCMEC/D3 cells were cultured at a density of 10.000 cells/mm^2^ on collagen-coated, 0.4 µm PTFE Transwells (Corning Inc., Corning, NY) for 6–7 days.

Integrity and functionality of the cultured cell monolayers were analyzed by measuring the transendothelial electrical resistance (TEER) using a volt/ohm meter (World Precision Instruments, Sarasota, FL/Millipore, Bedford, MA). The resistance of the collagen-coated inserts without cells was substracted from the resistance generated by the cell monolayers. Transcytosis assays were performed as TEER reached >50 ohms/cm^2^ [26]. TEER was measured routinely before and after the experiments with Ad vectors. Permeability assays based on the diffusion of lucifer yellow (LY; MW 457.25, Sigma-Aldrich) and 70 kDa dextran-FITC (De-FITC, Invitrogen) were performed as previously described [38]. Permeability coefficients were calculated according to published algorithms and compared to known permeability index of hCMEC/D3 cells (LY<1.33×10^−3^ cm/min, De-FITC<0.06×10^−3^ cm/min; [21].

Primary porcine brain capillary endothelial cells (PBCEC) were obtained or isolated according to established protocols [27]–[29]. Prior to transcytosis experiments the transwell inserts were coated with rat tail collagen (133 µg/ml, Sigma-Aldrich; 0.4 µm pore size polycarbonate filters, Corning Inc.) for 4 hrs and thawed cells were cultured at a density of 250.000 cells/filter for 5 days. Integrity of PBCEC monolayers were analyzed by measuring TEER using a voltohmeter in an electrode chamber. The cells were grown in serum-free DMEM/Ham`s F12 medium (Biochrom), supplemented with hydrocortisone (0.55 µM), L-glutamine (4.1 mM, Biochrom), penicillin-streptomycin (1%) and gentamycin (1%; Sigma-Aldrich). Transcytosis assays were performed in PBCEC cells as TEER reached >600 ohms/cm^2^.

### Ad Vector Production and Titration

Ad5-based E1-deleted first generation vectors were used, expressing EGFP under control of the hCMV promoter. AdFiberCys contains the peptide motif LIGGGCGGGID inserted in the fiber HI loop after amino acid 543 of the fiber protein sequence (size ∼110 nm; [19]. In AdHexonCys an alanine residue (amino acid position 273 in GenBank:AAQ19298.1) in the hypervariable region 5 of hexon was exchanged for a cysteine residue by mutating the corresponding alanine codon GCC (nt position 19658 in GenBank:AY339865.1) to TGC ([20]. All vectors were produced in E1-transcomplementing N52.E6 cells [39], followed by subsequent purification by CsCl gradients (step and isopycnic) and desalting by gel filtration (PD-10; Amersham, Buckinghamshire, UK). Cysteine-bearing vectors were purified under reducing conditions as described previously [19]. Vectors were stored at −80°C in PBS supplemented with 10% glycerol. Viral titers were determined by DNA-based slot blot procedure in A549 cells [19], [20].

### Covalent Chemical Coupling of Transferrin to Ad Vectors

Human apotransferrin was kindly provided by Prof. E. Wagner (Dept. of Pharmacy, Center for Drug Research, Ludwig-Maximilians-Universität Munich, Munich, Germany) and chemically coupled to Ad vectors as previously described [19], [20]. Briefly, to modify a surface amino group of apotransferrin with a thiol-reactive compound, apotransferrin was first incubated with NHS-PEG(3 kDa)-Mal crosslinker (Nektar Therapeutics, San Carlos, CA, PBS pH 7.3, molar ratio 1∶1, 4 hrs, RT), followed by purification with size exclusion chromatography (Äkta Purifier, Amersham). To couple the maleimide-activated apotransferrin molecules covalently to the cysteine-bearing Ad vectors, 10^11^ vector particles were incubated with the maleimide-modified proteins overnight at RT in an argon atmosphere (molar ratio vector surface cysteine:maleimide, 1∶30). Prior to all transduction or transcytosis experiments, iron was added to the cell culture media in order to enable the formation of iron-transferrin (hTf). For SDS-polyacrylamide gel electrophoresis and western blot analysis, transferrin was detected by horseradish peroxidase–labeled transferrin Ab (Bethyl Laboratories, Montgomery, TX). Virus proteins were identified by Ad5-fiber MAb (Ms IgG2a, DM3002; Acris GmbH, Hiddenhausen, Germany) or Ad5-hexon MAb (65H6, Ab Frontier, Seoul, Korea) using a horseradish peroxidase–labeled secondary Ab and the ECL kit (Thermo Fisher Scientific, Waltham, MA) for detection.

### Flow Cytometry Assays

K562 or hCMEC/D3 cells were seeded into 24-well-plates with or without the collagen coating (100.000–150.000 cells/well). After 24 hrs, 200–5000 physical vector particles per cell (pMOI) were added to medium (300–500 µl) for 3 hrs. The medium was filled up to 1 ml and incubated up to 24 hrs. hCMEC/D3 cells were washed with PBS and detached with PBS containing 50 mM EDTA. Both cell lines were centrifuged (350–500 g, 5 min), followed by resuspension in PBS/2%FCS/20 mM EDTA. For dextran uptake assays in K562 or hCMEC/D3 cells, 1 mg/ml of 70 kDa FITC-Dextran was incubated with the cells for 1 h at 37°C or 4°C followed by extensive washes with PBS. For hCAR cell surface labeling experiments, hCMEC/D3 cells were first detached by trypsin and incubated with hCAR MAb (Acris GmbH) for 30 min on ice, followed by subsequent washing and incubation with goat anti-mouse Alexa-488 (30 min, dark). To confirm that trypsin treatment did not impair hCAR antibody recognition, similar experiment was performed in A549 cells, known to have a high hCAR expression.

Flow cytometric analysis of virus-mediated EGFP expression, dextran uptake or hCAR expression was performed with a Becton-Dickinson FACSCalibur without gating (Becton-Dickinson, Franklin Lakes, NJ). For virus expression experiments, relative transduction efficiencies were calculated from the mean fluorescence intensity. For dextran uptake assays, fluorescence values obtained from samples on ice were substracted from those obtained at 37°C.

### Transwell Assays

Transcytosis assays were performed in Transwell-filter cultured hCMEC/D3 and PBCEC cells as TEER reached >50 ohms/cm^2^ or >600 ohms/cm^2^, respectively. Unmodified and modified Ad vectors (2×10^8^, 1×10^9^ or 5×10^9^ viral particles/filter/300 µl of full medium) were added to the apical side of the Transwells and incubated for 4 hrs at 37°C, 5% CO_2_. The medium from the basolateral side of the Transwell (800 µl) was collected, frozen (−80°C) and tested for the presence of transcytosed Ad particles by qPCR. To determine the intactness of the Ad particles, 20 µl of transcytosed vectors were diluted 10-fold to Tris-buffer (50 mM Tris, 1 mM MgCl_2_, pH 8), followed by treatment with DNAse (Sigma-Aldrich, 100 U/ml, 15 min, 37°C). Untreated vectors or heat (10 min, 95°C) and DNAse treated vectors were used as controls. Virus DNA was isolated with virus spin kit (Qiagen, Hilden, Germany) and analyzed by qPCR. For gene delivery studies, transcytosed vectors were immediately used to transduce 293 cells in order to avoid unnecessary freeze-thawing of the vectors. For transgene expression experiments in polarized hCMEC/D3 cells, after collecting the basolateral media, the cells were transferred to new plates and incubated further up to 24 hrs at 37°C, 5% CO_2_. The transduction efficiency was detected by fluorescence microscopy or fluorimetry (Twinkle LB970, Berthold Technologies, Bad Wildbad, Germany) after detaching the cells by scraping and diluting them to PBS-EDTA.

### Quantitative Real-Time PCR

To determine the number of transcytosed vector particles, qPCR was performed from basolateral media using either Ad fiber or E4 primers and appropriate standards (fiber primers, AdFiberCys/AdHexonCys vectors: sense 5-GCTACAGTTTCAGTTTTGGCTG-3, reverse 5-GTTGTGGCCAGACCAGTCCC-3, amplicon length 386 bp; E4 primers, AdFiberCys; sense 5-TAGACGATCCCTACTGTACG-3, reverse 5-CCGGACGTAGTCATATTTCC-3, amplicon length 96 bp). The corresponding Ad copy number was determined by standard curve of linearized plasmid pGS66 (r^2^>0.995, E  =  >98%), containing the Ad vector genome (Schiedner et al., 2000). Five to ten microliters of virus spin kit isolated viral DNA (Qiagen) was added to a total of 25 µl reaction mixture consisting of primers (10 pmol) and 10 µl of 2x SYBR Green Master Mix (Stratagene, Agilent Technologies, Santa Clara, CA). Amplification was detected by MxPro3005p on 96-well plates (Stratagene, Agilent Technologies). Following denaturation at 95°C for 10 min, cycling conditions were 95°C for 30s, 60°C for 30s, 72°C for 30s for 40 cycles. The formation of primer dimers was monitored by gel electrophoresis of the PCR products and by the melting curves with MxPro3005p software. Total copy numbers of input vectors detected were between 60–90%. Data presented is shown either as copy numbers of the detected fiber or E4 gene or as the percentage of transcytosed virus from the applied input virus.

### Gene Transfer Assay for Transcytosed Vectors

The functional integrity of the transcytosed vector particles was tested by examining their gene transfer efficiency in hTf-receptor positive 293 cells. For each experiment, 100–150 µl of collected basolateral media was used to transduce 293 cells for 16 hrs. Untransduced control cells were used to substract the autofluorescence and set-up the imaging settings. By fluorescence microscopy (Zeiss Axiovert 25, 20x/0.4 objective, Carl Zeiss AG, Oberkochen, Germany) and ImageJ [40] EGFP expressing cells were counted from six to eight random areas and compared to total amount of the cells in the same areas (n = 400–500 cells). Transduction units (t.u.) were calculated from the total sample volumes.

### Statistical Analysis

The significance of the data was determined by Student’s t-test or Wilcoxon-Mann-Whitney U-test (SPSS). Box plot data was done by R software (R Development Core Team, Vienna, Austria).
